# Expression of tumor necrosis factor-like weak inducer of apoptosis and fibroblast growth factor-inducible 14 in patients with polymyositis and dermatomyositis

**DOI:** 10.1186/ar4454

**Published:** 2014-01-27

**Authors:** Qing-Lin Peng, Xiao-Ming Shu, Xiao-Lan Tian, Xin Lu, Guo-Chun Wang

**Affiliations:** 1 Department of Rheumatology, China-Japan Friendship Hospital, Ying Hua East Road, Chao Yang District, Beijing 100029, China; 2 Graduate School of Peking Union Medical College, Beijing, China

## Abstract

**Introduction:**

The aim of this study was to investigate the expression of tumor necrosis factor-like weak inducer of apoptosis (TWEAK) and its receptor fibroblast growth factor-inducible 14 (Fn14) in patients with polymyositis (PM) and dermatomyositis (DM), and their relation to clinical manifestations.

**Methods:**

Serum levels of TWEAK were detected in 98 PM/DM patients and 37 healthy controls by using the ELISA method. Total RNA isolated from fresh-frozen muscle tissue samples of 36 PM/DM patients and 10 healthy controls were used for analyzing the mRNA levels of TWEAK and Fn14 by quantitative reverse transcription polymerase chain reaction (RT-PCR). Immunofluorescence staining of TWEAK and Fn14 was conducted on muscle biopsy specimens from 23 PM/DM patients and seven healthy controls.

**Results:**

Serum levels of TWEAK were significantly decreased in the PM/DM patients compared to those in the healthy controls (*P* < 0.001), and serum TWEAK levels negatively correlated with serum CD163 levels in PM/DM patients (r = -0.49, *P* < 0.001). The expression of Fn14 mRNA was significantly increased in the muscle tissue of PM/DM patients than in the muscle tissue of healthy controls (*P* < 0.01), whereas the expression of TWEAK mRNA in PM/DM patients was not statistically different from that of the healthy controls (*P* > 0.05). Fn14 mRNA levels in muscle tissue positively correlated with muscle disease activity (r = 0.512, *P* < 0.01). Patients with oropharyngeal dysphagia had significantly higher Fn14 mRNA levels than patients without oropharyngeal dysphagia (*P* < 0.05). The results of immunofluorescence staining showed that 19 out of 23 PM/DM patients were TWEAK-positive, and 20 out of 23 PM/DM patients were Fn14-positive. No detectable expressions of TWEAK or Fn14 were observed in the healthy controls.

**Conclusions:**

TWEAK-Fn14 axis may be involved in the pathogenesis of PM/DM. Further understanding of TWEAK-Fn14 function in PM/DM may help to define therapeutic targets for PM/DM.

## Introduction

Idiopathic inflammatory myopathies (IIM) are a heterogeneous group of muscle disorders characterized by symmetrical proximal muscle weakness, decreased muscle endurance, and inflammatory infiltrates in skeletal muscle tissue [1,2]. Polymyositis (PM) and dermatomyositis (DM) are two common subsets of IIM. Although the pathological mechanism of IIM remains unclear, recent studies suggest that an interplay between adaptive immune, innate immune, and nonimmune mechanisms may be responsible for the damage and dysfunction that occur in myopathic muscle tissue [3]. Multiple players - such as adaptive immune cells [4], cytokines and chemokines [5-7] - may be involved in the pathogenesis of IIM.

Tumor necrosis factor-like weak inducer of apoptosis (TWEAK) is a recently identified pro-inflammatory cytokine belonging to the TNF superfamily [8]. TWEAK has emerged as a multifunctional cytokine that regulates multiple cellular responses, including pro-inflammatory activity; angiogenesis; and cell proliferation, differentiation, migration and apoptosis [9]. The only known signaling receptor for TWEAK is fibroblast growth factor-inducible 14 (Fn14), which was first recognized by differential display technique [10]. TWEAK exerts pleiotropic functions through binding to Fn14 and activating downstream signaling pathways. Fn14 is a type I transmembrane protein and is highly inducible by various growth factors, including epidermal growth factor (EGF), platelet-derived growth factor (PDGF), and vascular endothelial growth factor (VEGF). Fn14 is predominantly expressed on the surface of epithelial cells, endothelial cells and other non-hematopoietic cells [9,11].

TWEAK was found to activate nuclear factor-кB (NF-кB) and mitogen-activated protein kinase (MAPK) signaling pathways and to reduce MyoD levels in cultured myoblasts [12,13], thereby inducing the proliferation of myoblasts and inhibiting their differentiation into myotubes [13]. In addition, several studies have demonstrated that the TWEAK-Fn14 axis plays a crucial role in muscle atrophy [14-16]. According to the findings of Bhatnagar *et al.*, TWEAK causes myotube atrophy by stimulating the activity of the ubiquitin-proteasome system, autophagy and caspases [15]. Coincidentally, Alger *et al.* reported the expression of autophagic genes (beclin and LC3) at the protein level in PM/DM muscle biopsy specimens but not in healthy controls, suggesting the potential role of autophagy in mediating muscle fiber death in myositis [17]. Considering the role of TWEAK in stimulating autophagy and impairing myogenesis, we hypothesized that the TWEAK-Fn14 axis may be involved in the pathomechanism of PM/DM and set out to study whether TWEAK-Fn14 participates in the pathogenesis of PM/DM. Additionally, increased levels of TWEAK were found in many types of autoimmune diseases, such as rheumatoid arthritis [18], systemic sclerosis [19], and systemic lupus erythematosus [20], suggesting a crucial role of TWEAK in autoimmune disorder. However, information about TWEAK-Fn14 expression in PM and DM is limited.

Therefore, in order to further explore the role of TWEAK in the pathogenesis of PM/DM, we investigated the levels of circulating TWEAK in sera and the expression of TWEAK and Fn14 in the muscle tissues of patients with PM/DM at the mRNA and protein levels.

## Methods

### Patients

From the inpatients and outpatients who visited China-Japan Friendship Hospital between 2009 and 2012, 98 patients with PM/DM were recruited for this study. Their diagnoses of PM/DM were based on the Bohan and Peter criteria [21]. Additionally, 37 healthy, age-matched and sex-matched volunteers were selected to be the healthy control group during the same time period. Muscle tissues from 10 trauma patients without muscle disease were used as normal controls for examination of TWEAK and Fn14 in muscle tissue. For each individual patient, serum sampling and disease activity evaluation were done at the same time. For patients who carried out muscle biopsy in our department, muscle biopsy was also done at the time of serum sampling. This study was performed with the approval of the Human Ethics Board of China-Japan Friendship Hospital (Beijing, China). Written informed consent was obtained from all participating individuals.

### Measurement of serum TWEAK levels and serum CD163 levels

Fresh venous blood samples were centrifuged shortly after clot formation, and serum samples were collected. All samples were stored at -70°C before use. TWEAK was detected in the sera of 98 PM/DM patients and 37 healthy controls using a commercially available ELISA kit (Bender MedSystems, Vienna, Austria). The assays were performed according to the manufacturer’s protocol. Briefly, 100 μl distilled water were added to all standard wells, blank wells and sample wells, then 50 μl of each sample, in duplicate, were added to the designated wells. After incubating at room temperature for 3 hours, the plates were washed and incubated with 100 μl of 3,3′,5,5′-tetramethylbenzidine (TMB) substrate solution. About 10 minutes later, stop solution was added, and absorbance was measured using the ELISA reader at 450 nm with 630 nm as the reference wavelength. A standard curve for each assay was generated, and serum TWEAK concentration was calculated. The assays for each sample were performed three times.

Serum CD163 levels were measured by using a quantitative ELISA kit (R&D Systems, Minneapolis, MN, USA). The assays were performed according to the manufacturer’s protocol. Each sample was measured three times.

### Assessment of disease activity

Disease activity was measured by the 2005 myositis disease activity assessment tool (MDAAT) established by the International Myositis Assessment and Clinical Studies (IMACS) group [22], which consists of the myositis disease activity assessment visual analog scales (MYOACT) and the myosits intention-to-treat index (MITAX). In the previous studies, our data showed that the MYOACT could be used to reliably evaluate disease activity in Chinese patients with PM/DM [23]. Therefore, we used MYOACT to evaluate the disease activity of PM/DM patients following the guidelines for physician scoring of disease activity.

### Real time RT-PCR

Total RNA was isolated from fresh-frozen muscle tissue using TRIzol reagent (Invitrogen, Carlsbad, CA, USA) according to the manufacturer’s protocol. Two μg of total RNA was reverse transcribed using the GoTaq® 2-Step RT-qPCR system (Promega, Madison, WI, USA) according to the manufacturer’s protocol. Fluorescent quantitative reverse transcription polymerase chain reaction (RT-PCR) was performed using the ABI PRISM 7500 system (Applied Biosystems, Foster City, USA) according to standard methods. The following primers were used for amplifying TWEAK, Fn14 and glyceraldehyde-3-phosphate dehydrogenase (GAPDH): TWEAK, forward: 5′-CCCTGCGCTGCCTGGAGGAA-3′, reverse: 5′-AGACCAGGGCCCCTCAGTGA-3′; Fn14, forward: 5′-CCAAGCTCCTCCAACCACAA-3′, reverse: 5′-TGGGGCCTAGTGTCAAGTCT-3′; GAPDH forward: 5′-ATCACCATCTTC CAGGAGCGA-3′, reverse: 5′- CCTTCTCCATGGTGGTGAAGAC-3′. Real time RT-PCR for TWEAK, Fn14 and GAPDH was conducted in triplicate for each sample. The *GAPDH* gene was used as an internal control: 2^−Δ CT^ was used to compute the expression value of target genes.

### Hematoxylin and eosin staining of muscle sections

H&E staining was applied on serial cryostat muscle biopsy sections according to the following steps. The muscle sections were stained with Harris Hematoxylin (Sigma-Aldrich, St Louis, MO, USA) for 5 minutes. After rinsing in running tap water for 20 minutes, the muscle sections were immersed in working eosin solution for 2 minutes. At last, the muscle sections underwent the dehydration of gradient ethanol, the transparency of dimethylbenzene, and then were mounted with Canada balsam (Sigma-Aldrich).

### Immunofluorescence staining of muscle sections

8-μm-thick unfixed cryostat muscle sections were collected from diagnostic muscle biopsies. Anti-human TWEAK polyclonal antibodies (R&D systems) and anti-human Fn14 monoclonal antibodies (R&D systems) were used as primary antibodies for detecting TWEAK and Fn14 at a working concentration of 15 μg/ml. Rhodamine-conjugated anti-goat IgG (Santa Cruz Biotechnology, Santa Cruz, CA, USA) was used as a secondary antibody. In summary, immunofluorescence staining was conducted according to the following steps: muscle sections were fixed with pre-cooled acetone at -20°C for 30 minutes and then rinsed four times in phosphate-buffered solution. Then, the tissue sections were blocked in 10% normal serum with 1% BSA in tris-buffered saline (TBS) for 2 hours at room temperature. After draining for a few seconds, primary antibodies diluted in TBS were applied to the tissue sections. After incubating overnight at 4°C, the tissue sections were treated with rhodamine-conjugated anti-goat IgG. This was followed by washing, mounting on glass slides, and visualization of fluorescence with an Olympus fluorescent microscope. An isotype-matched, non-binding antibody (normal goat IgG: sc-2028, Santa Cruz Biotechnology) was used as a negative control for the primary antibody. Two sections per tissue sample were used for immunofluorescence staining analysis.

When double staining was carried out, anti-human TWEAK goat polyclonal antibodies (R&D systems) and anti-human Fn14 rabbit monoclonal antibodies (Abcam, Cambridge, UK) were used as primary antibodies for detecting TWEAK and Fn14, and Rhodamine-conjugated donkey anti-goat IgG and FITC-conjugated donkey anti-rabbit IgG (both from Santa Cruz Biotechnology) were used as secondary antibodies.

### Statistical analysis

Statistical analysis was performed using GraphPad Prism V.4.03 (GraphPad Software, San Diego, USA) and SPSS V.16.0 (SPSS, Chicago, USA). Quantitative variables were described as mean ± SD. Nonparametric distribution data were expressed as median value and IQR, and the data of unpaired samples were analyzed using the Mann-Whitney *U*-test. Spearman’s correlation analysis was used to test for correlation. The Wilcoxon signed rank test was used on paired data when appropriate. A *P*-value equal to or less than 0.05 was considered statistically significant.

## Results

### Clinical characteristics of PM/DM patients

The clinical features presented by the 98 patients who were recruited for this study are summarized in Table 1. According to criteria established by Bohan and Peter [21], 41 patients were diagnosed with PM, and the other 57 patients were diagnosed with DM. The mean age of these patients at onset of PM/DM was 50.6 years. There were more females than males in the cohort. The mean duration of disease was 5.3 years (range from 1.3 to 28.0 years). Twenty patients were examined as anti-Jo-1 antibody-positive at the time of blood sampling for the present study.

**Table 1:** Demographic and clinical features of PM/DM patients and healthy controls

	**PM/DM patients**	**Healthy controls**
Age of onset, mean ± SD (range) years	50.6 ± 17.4 (21.5 to 75.0)	51.4 ±9.4 (27 to 70)
Number of females/number of males	77/21	27/10
Number of PM patients/number of DM patients	41/57	NA
Duration of disease, mean ± SD (range) years	5.3 ± 7.6 (1.3 to 28.0)	NA
Clinical features, number of patients affected (% of cohort)		
Interstitial lung disease	50 (51.0%)	NA
Oropharyngeal dysphagia	35 (35.7%)	NA
Raynaud’s phenomenon	20 (20.4%)	NA
Mechanic’s hands	12 (12.2%)	NA
Arthritis	45 (45.9%)	NA
Anti-Jo-1-positive at time of blood sampling	20 (20.4%)	NA
Levels of creatine kinase at initial visit, mean ± SD	1437.2 ± 1536.5	NA

PM, polymyositis; DM, dermatomyositis; NA, not applicable.

In our cohort, 41 patients were newly diagnosed in our department, while the other 57 patients had been diagnosed with PM/DM and received different treatments before they were referred to our hospitial. The treatments received by the patients in our cohort varied according to the severity of their disease. All patients received corticosteroid at doses between 0.5 mg/kg and 1 mg/kg as part of their initial therapy after their diagnosis of PM/DM. Meanwhile, about 80% of our patients (78 out of 98) also received at least one or more immunosuppressants, among which were methotrexate, cyclophosphamide, azathioprine, intravenous immunoglobulin, hydroxychloroquine and mycophenolate mofetil.

### Lower serum levels of TWEAK in patients with PM/DM

**Figure 1:**
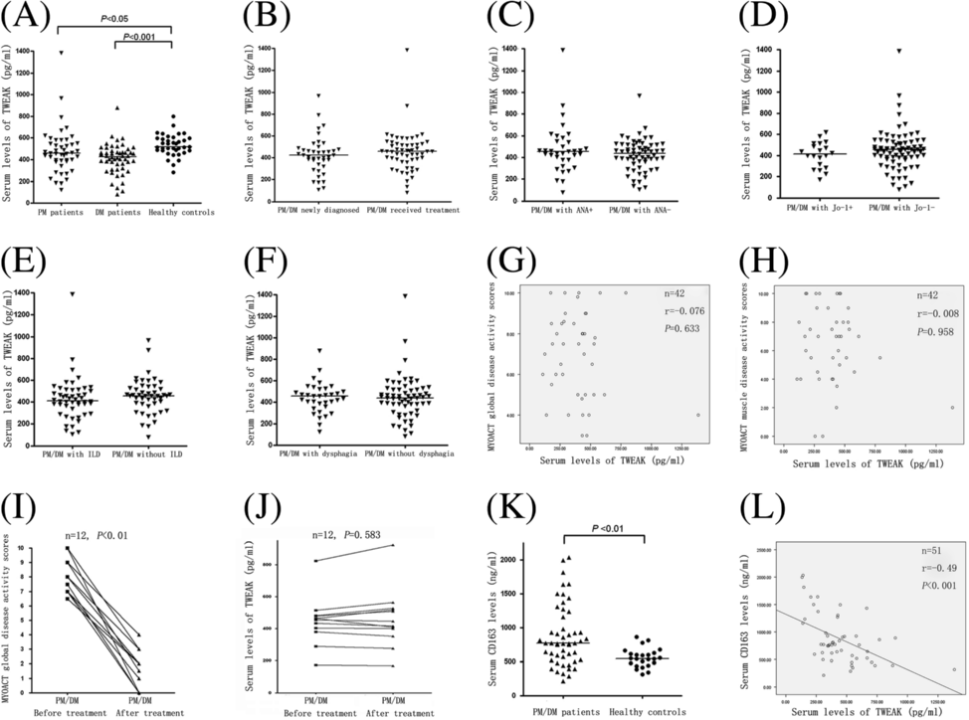
**Serum levels of tumor necrosis factor-like weak inducer of apoptosis (TWEAK) in patients with polymyositis (PM)/dermatomyositis (DM) and in healthy controls.** Serum TWEAK levels were determined by a specific ELISA. TWEAK levels in healthy subjects were compared to patienta with PM or DM **(A)**. There was no statistical difference in serum TWEAK levels between patients previously untreated and patients who received treatments **(B)**. No significant differences were observed when TWEAK levels were compared in patients with PM/DM sub-grouped based on antinuclear antibody (ANA) positivity **(C)**, Jo-1 positivity **(D)**, presence of interstitial lung disease (ILD) **(E)** and presence of oropharyngeal dysphagia **(F)**. Correlation of serum levels of TWEAK with myositis disease activity assessment visual analog scales (MYOACT) global disease activity **(G)** and MYOACT muscle disease activity **(H)** were analyzed. Twelve newly diagnosed PM/DM patients had significantly decreased MYOACT global disease scores after treatment **(I)**, however, no significant difference was observed in the patients’ serum TWEAK levels before and after treatment **(J)**. Serum CD163 levels were determined by a quantitative ELISA. Serum CD163 levels were significantly higher in PM/DM patients compared to healthy controls **(K)**, and serum CD163 levels were found to be negatively correlated with serum TWEAK levels in PM/DM patients **(L)**. The data for unpaired samples were analyzed by using the Mann-Whitney *U*-test. Spearman’s correlation analysis was used to test for correlation. The Wilcoxon signed rank test was used on paired data. Horizontal bars indicate median levels.

Serum levels of TWEAK were measured in 98 patients with PM/DM. The ELISA kit used in this study can detect all circulating TWEAK in sera. We found that the median value of serum TWEAK concentration in PM/DM patients was 442 pg/ml (range 81 to 1385 pg/ml), while the median value of that in healthy controls was 516 pg/ml (range 282 to 797 pg/ml). Therefore, serum levels of TWEAK were significantly lower in PM/DM patients compared to that in healthy controls (*P* <0.001). In addition, serum levels of TWEAK showed no statistical difference between PM and DM patients (*P* >0.05), as shown in Figure 1A. Among the 98 PM/DM patients in our cohort, 41 patients were newly diagnosed therefore the serum samples of these patients were collected before they received treatment. The other 57 patients were referred to our department, and they had received corticosteroids and other immunosuppressive treatment before we collected serum samples from them. However, we did not find a significant difference in serum TWEAK levels between these two groups of PM/DM patients (*P* >0.05), as shown in Figure 1B.

We analyzed the correlation between serum TWEAK levels and clinical parameters. However, no significant differences were observed when TWEAK levels were compared in patients with PM/DM sub-grouped based on ANA positivity (Figure 1C), Jo-1 positivity (Figure 1D), presence of interstitial lung disease (ILD) (Figure 1E) and presence of oropharyngeal dysphagia (Figure 1F). What is more, no correlation was found between serum TWEAK levels and MYOACT global disease activity scores in 42 PM/DM patients (Figure 1G) or MYOACT muscle disease activity (Figure 1H). In our study cohort, 12 patients were newly diagnosed, and their serum samples before and after treatment were collected. After treatment, all the 12 patients were considered as in remission stage and their MYOACT global disease scores were significantly decreased (*P* <0.01), as shown in Figure 1I. However, by applying the Wilcoxon signed rank test, no significant difference was observed in the patients’ serum TWEAK levels before and after treatment (*P* >0.05), as shown in Figure 1J.

As some studies have revealed that CD163 expressed by monocytes could act as a TWEAK scavenger in pathological conditions [24], we detected the serum levels of CD163 in PM/DM patients and healthy controls. Interestingly, we found that serum CD163 levels were significantly higher in PM/DM patients (median value/IQR 770.0 ng/ml, range 212.6 to 2033.1 ng/ml) compared to healthy controls (median value/IQR 544.0 ng/ml, range 313.0 to 859.8 ng/ml) (*P* <0.01) (Figure 1K), and serum CD163 levels were found to be negatively correlated (*r* = −0.49, *P* <0.001) with serum TWEAK levels in PM/DM patients (Figure 1L).

### Increased expression of Fn14 mRNA in muscle tissue of patients with PM/DM

**Figure 2:**
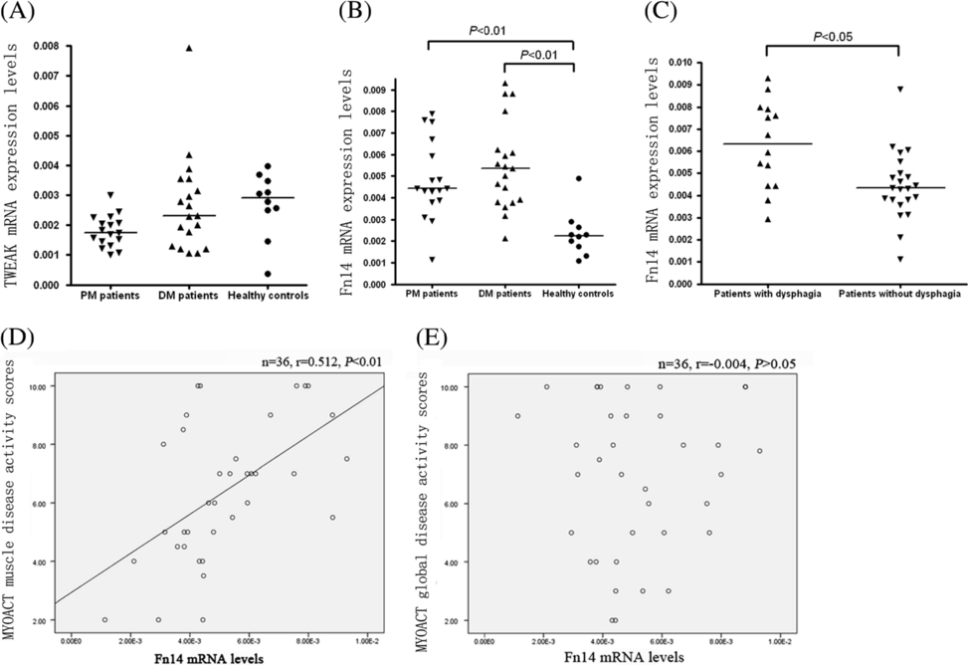
**Comparison of tumor necrosis factor-like weak inducer of apoptosis (TWEAK) and Fn14 mRNA levels of polymyositis (PM)/dermatomyositis (DM) and of healthy controls.** Total RNA was isolated from fresh-frozen muscle tissue using TRIzol reagent, and fluorescent quantitative reverse transcription polymerase chain reaction (RT-PCR) was performed. The glyceraldehyde-3-phosphate dehydrogenase (*GAPDH*) gene was used as an internal control. 2^−Δ CT^ was used to compute the expression values of the target genes. **(A)** TWEAK mRNA levels in muscle tissue of PM, DM and healthy controls. **(B)** Fn14 mRNA levels in muscle tissue of PM, DM and healthy controls. **(C)** Fn14 mRNA levels were compared in PM/DM patients sub-grouped based on the presence of oropharyngeal dysphagia. **(D)** Correlation of Fn14 mRNA levels with myositis disease activity assessment visual analog scales (MYOACT) muscle disease activity scores. **(E)** Correlation of Fn14 mRNA levels with MYOACT global disease activity scores. The data of unpaired samples were analyzed by using the Mann-Whitney *U*-test. Spearman’s correlation analysis was used to test for correlations. Horizontal bars indicate median levels.

The expression of TWEAK and Fn14 mRNA in the muscle tissue of 17 PM, 19 DM patients and of 10 healthy controls was analyzed by real time RT-PCR. The diagnoses of the 36 PM/DM patients were confirmed by pathological diagnoses of muscle biopsies. The expression of TWEAK mRNA in the muscle tissue of patients with PM/DM was not significantly different from that of the healthy controls (*P* >0.05) (Figure 2A). However, the expression of Fn14 mRNA was significantly higher in the muscle tissue of PM/DM patients than in the healthy controls (*P* <0.01) (Figure 2B). The Fn14 mRNA levels showed no significant difference between PM and DM patients (*P* >0.05) (Figure 2B). Interestingly, we found that patients with oropharyngeal dysphagia had significantly higher Fn14 mRNA levels than the patients without oropharyngeal dysphagia (Figure 2C). In addition, the Fn14 mRNA levels correlated with MYOACT muscle disease activity scores (*r* = 0.512, *P* <0.01), but did not correlate with MYOACT global disease activity score (*P* >0.05), as shown in Figure 2D and Figure 2E respectively.

### High expression of TWEAK and Fn14 protein in PM/DM muscle biopsies

**Figure 3:**
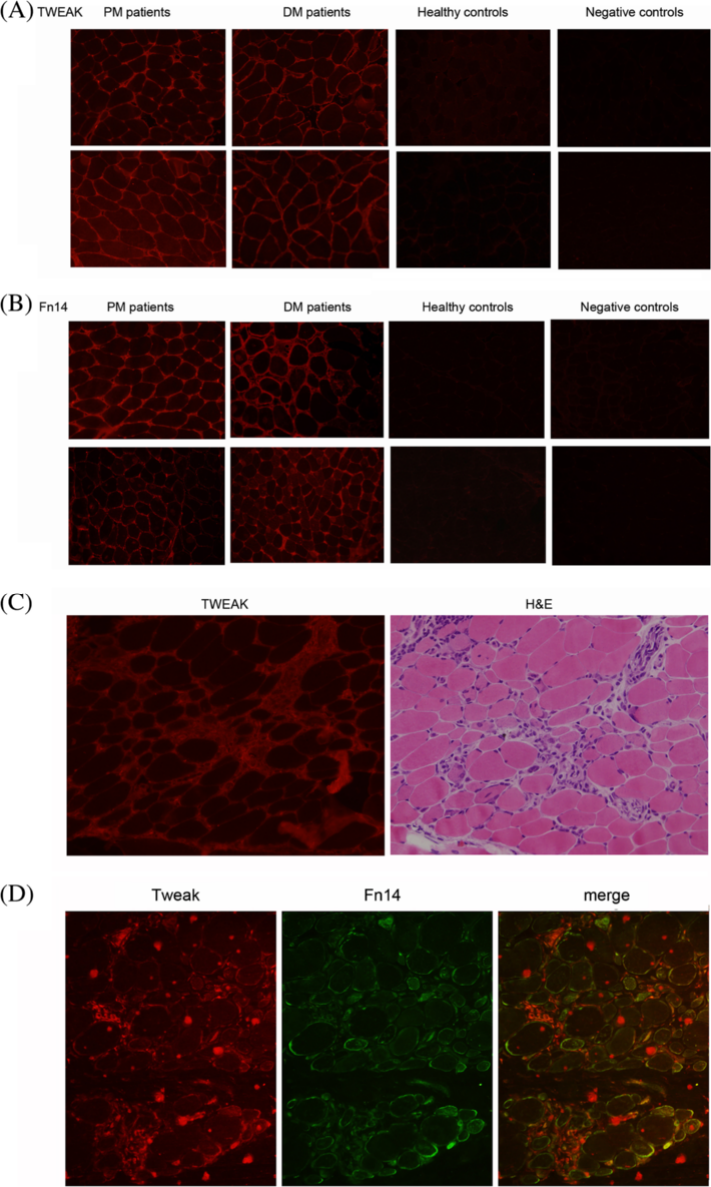
**Expression of tumor necrosis factor-like weak inducer of apoptosis (TWEAK) and Fn14 in patients with polymyositis (PM)/dermatomyositis (DM) and in healthy controls. (A)** Muscle biopsies from patients with PM/DM showed significantly higher levels of the TWEAK protein compared to those of healthy controls. Anti-human TWEAK polyclonal antibodies were used as the primary antibody for detection at a working concentration of 15 μg/ml. Rhodamine-conjugated anti-goat IgG was used as a second antibody. **(B)** In PM/DM biopsies, expression of the Fn14 protein was remarkably higher than that of healthy controls. Anti-human Fn14 monoclonal antibodies were used as the primary antibody for detection at a working concentration of 15 μg/ml. Rhodamine-conjugated anti-goat IgG was used as a second antibody. Each muscle section was analyzed in duplicate, and results from one representative experiment are shown. Negative controls were conducted by staining muscle biopsies with isotype control antibody, and no obvious immunofluorescence signal was observed in negative controls. **(C)** Serial muscle biopsy sections of PM/DM patients were applied for TWEAK immunofluorescence staining and H&E staining, and a representative picture of the same type of field is shown. **(D)** Muscle biopsy sections of PM/DM patient were applied for TWEAK and Fn14 immunofluorescence staining and a representative staining is shown. To ensure comparability, images were obtained using identical exposure settings for each channel. The TWEAK immunoreactivity is predominantly observed around the infiltrating monocytes, and a mild expression of TWEAK on the sarcolemma of muscle fibers is also visible. While Fn14 are mainly expressed on the sarcolemma of muscle fibers. Therefore, TWEAK and Fn14 are frequently found to be coexpressed in the same myofibers on overlay images. Magnification is 200 ×.

We analyzed the expressions of TWEAK and Fn14 in the muscle biopsies of 13 patients with DM, 10 patients with PM, and 7 healthy controls using immunofluorescence staining. The diagnoses of the 23 PM/DM patients received pathological confirmation. As shown in Table 2, strong expressions of TWEAK were observed in 8 of the 10 PM patients and in 11 of the 13 DM patients; none of the healthy controls showed obvious expression of TWEAK in muscle biopsies. Similarly, the results of immunofluorescence staining showed remarkable expression of Fn14 in 8 of the 10 PM patients and in 12 of the 13 DM patients; there was no significant expression of Fn14 in any of the healthy controls. Additionally, all patients who were Fn14-negative also showed no detectable TWEAK expression. However, the muscle biopsy of one DM patient stained positive for Fn14 but stained negative for TWEAK. Figure 3A and Figure 3B shows representative results of immunofluorescence staining of the muscle biopsies. Muscle biopsies from the healthy controls showed no significant TWEAK or Fn14 expression. And no obvious immunofluorescence signal was observed in PM/DM muscle biopsies staining with isotype control antibody.

**Table 2:** PM/DM patients with positive immunofluorescence staining for TWEAK and Fn14

	**PM patients**	**DM patients**	**Healthy controls**
TWEAK positive (number/number assessed)	8/10	11/13	0/7
Fn14 positive (number/number assessed)	8/10	12/13	0/7

TWEAK, tumor necrosis factor-like weak inducer of apoptosis; PM, polymyositis; DM, dermatomyositis.

Additionally, in order to analyze TWEAK expression relative to muscle pathology, we conducted H&E staining on serial muscle biopsy sections. Figure 3C showed the same type of field on TWEAK immunofluorescence staining and H&E staining. We observed a remarkably strong expression of TWEAK in the field where abundant infiltrating monocytes existed. TWEAK immunoreactivity was also observed around the sarcolemma of muscle fibers partially invaded or surrounded by monocytes, and membrane TWEAK staining was also visible in a large number of fibers that were not near inflammatory cells.

To further demonstrate the localization of the TWEAK and Fn14 protein, we carried out double staining for TWEAK and Fn14. As shown in Figure 3D, the expression of TWEAK was dramatically observed around the infiltrating monocytes, and a mild expression of TWEAK on the sarcolemma of muscle fibers was also visible, whereas Fn14 was mainly expressed on the sarcolemma of muscle fibers. We therefore found that TWEAK and Fn14 are frequently coexpressed in the same myofibers on overlay images (Figure 3D, merge). This was probably due to the soluble TWEAK protein secreted by inflammatory cells that could bind to the Fn14 expressed on the sarcolemma of muscle fibers.

## Discussion

In this study, we found a significantly lower level of serum TWEAK and a statistically higher level of serum CD163 in PM/DM patients compared to those in healthy controls. Serum CD163 levels were found to be negatively correlated with serum TWEAK levels in PM/DM patients. Relative quantification mRNA analysis showed that Fn14 mRNA expression in the muscle tissue of PM/DM patients was higher than in healthy controls, and Fn14 mRNA levels positively correlated with MYOACT muscle disease activity scores. However, there was no significant difference between the TWEAK mRNA expressions in the muscle tissue of PM/DM patients and of healthy controls. We also examined expressions of TWEAK and Fn14 in muscle biopsy specimens at the protein level and found that TWEAK and Fn14 expressions were significantly increased in PM/DM patients.

Serum levels of TWEAK were found to be elevated in rheumatoid arthritis [25] and systemic sclerosis [19]. In patients with systemic lupus erythematosus, urinary TWEAK level was found to be increased [26], but controversial results were obtained when serum TWEAK levels in systemic lupus erythematosus were investigated [26,27]. These findings suggest that TWEAK probably plays an important pathogenic role in autoimmune disorders [28,29]. However, in our study, we found that serum levels of TWEAK in PM/DM patients were significantly lower compared to those in healthy controls. We analyzed serum levels of TWEAK in 98 patients with PM/DM, and each sample was measured three times. Therefore, possible bias resulting from sample size is reduced. The interesting finding of lower serum TWEAK levels in PM/DM patients prompted us to find a reasonable explanation. Recently, it has been shown that CD163 can specifically bind and neutralize TWEAK [24] and that CD163-expressing monocytes/macrophages are able to bind and internalize exogenous TWEAK [30]. Taking into account molecular interaction between TWEAK and CD163 molecules, in the present study we evaluated serum CD163 levels in PM/DM patients, and we found a negative correlation between serum levels of TWEAK and CD163. These results probably gave a good explanation for the lower serum levels of TWEAK in PM/DM patients. Although the role of the serum TWEAK- CD163 interaction in PM/DM pathogenesis remains to be elucidated, our findings suggest a potential role of serum TWEAK and CD163 molecules in PM/DM.

Our further research showed that TWEAK expression at the protein level was remarkably increased in the muscle tissue of PM/DM patients, but TWEAK expression at the mRNA level showed no statistical difference with that of healthy controls. As we know, leukocytes are the major source of TWEAK [9], and muscle cells are unlikely to express the TWEAK protein. Because most of the mRNA extracted from muscle tissues was derived from muscle cells, it is reasonable that TWEAK mRNA levels showed no difference between PM/DM patients and healthy controls. In addition, we analyzed TWEAK immunofluorescence staining relative to H&E staining on serial biopsy sections. We observed a remarkably strong expression of TWEAK in the field where abundant infiltrating monocytes existed. Moreover, we carried out double staining for TWEAK and Fn14 on muscle biopsy sections to demonstrate the localization of the TWEAK and Fn14 protein, and the result showed that the TWEAK immunoreactivity was predominantly observed around the infiltrating monocytes, and a mild expression of TWEAK on the sarcolemma of muscle fibers was also visible, whereas Fn14 was mainly expressed on the sarcolemma of muscle fibers. Therefore, it is probable that the marked evidence of TWEAK protein in the muscle tissues of PM/DM patients was not due to expression by muscle cells but was a product of locally infiltrating leukocytes, which was secreted by inflammatory cells and bound to the Fn14 expressed on the sarcolemma of muscle fibers.

In the study of Mustonen *et al.*, TWEAK and Fn14 expression were assessed in experimental models of cardiac remodeling, and the results showed that the mRNA and protein levels of Fn14 were significantly upregulated, whereas the TWEAK mRNA and protein levels remained almost unchanged [31]. Therefore, their study challenged the previous assumptions of TWEAK expression in response to injury, and provided new insights into the role of ligand-independent Fn14 signaling in cardiac remodeling [31]. In contrast to cardiac remodeling, our study showed increased expression of TWEAK protein in locally infiltrating leukocytes and upregulated expression of Fn14 mRNA and protein on the sarcolemma of muscle fibers. The present study therefore suggested a possible role of TWEAK-Fn14 signaling in the pathogenesis of PM/DM.

Aberrant expression of TWEAK and/or Fn14 has been found to be linked with deleterious pathogenic effects in autoimmune diseases [9]. The TWEAK cytokine and its receptor, Fn14, are identified as critical regulators of skeletal muscle mass [9,16]. It has been proposed that TWEAK causes Fn14 trimerization and subsequent activation of several signaling proteins including TRAF6, transforming growth factor-β activated kinase 1, I kappa B kinase, and MAPK leading to the activation of various transcription factors followed by gene expression [9,32-34]. Moreover, Mittal *et al.* reported that TWEAK could suppress the regeneration and growth of myofibers after injury and that the expression of both TWEAK and Fn14 were increased in skeletal muscle after injury caused by intramuscular injections of cardiotoxin [35]. What is more, it is reported by Girgenrath *et al.* that Fn14 is highly expressed on muscle progenitor cells and TWEAK could inhibit myogenic differentiation [36]. Recently, it has been demonstrated by Enwere *et al.* that high concentrations of TWEAK could activate the canonical NF-кB pathway and impair myogenesis [37]. Therefore, the TWEAK-Fn14 axis may contribute to muscle atrophy by impairing skeletal muscle formation. It is worth mentioning that in PM/DM, impairment of muscle regeneration is also a critical characteristic of disease progression. Our study found that Fn14 expression was higher at both mRNA and protein levels in the muscle tissue of PM/DM patients. What is more, we analyzed the relationship between TWEAK and Fn14 expressions and clinical parameters, and found that patients with oropharyngeal dysphagia had significantly higher Fn14 mRNA levels than patients who did not suffer from oropharyngeal dysphagia. Furthermore, our study showed a moderate correlation between the Fn14 mRNA levels and muscle disease activity in PM/DM patients. Additionally, TWEAK protein levels were also significantly elevated in the muscle tissue of PM/DM patients, although their derivation needs to be further confirmed. Taken together, our findings suggest that the TWEAK-Fn14 axis is probably involved in the pathogenesis of PM/DM.

As stated by Morosetti *et al.*, dysregulation of the TWEAK-Fn14 axis in inclusion-body myositis (IBM) muscle may play a dual role in the pathogenesis of IBM by inducing muscle wasting and inhibiting regenerative myogenesis [38]. In their study, TWEAK and Fn14 expression in the muscle tissues of six DM patients, six PM patients and three healthy controls was analyzed by immunohistochemistry. Results showed that only muscle fibers that were invaded or surrounded by monocytes and degenerating fibers next to inflammatory infiltrates showed increased membrane TWEAK and Fn14 immunoreactivity [38]. Our findings seem to be inconsistent with their results. However, in our study, a larger number of patients were analyzed for TWEAK and Fn14 expression. More importantly, all the patients whose biopsies were examined for TWEAK and Fn14 expression by immunofluorescence staining received confirmed pathological diagnoses. On the other hand, whether these contradictory findings are a result of ethnic diversity needs to be verified by further studies.

To our knowledge, our study was the first to systematically investigate the expression of TWEAK and Fn14 in a large cohort of PM/DM patients. The strength of our study is that all the PM/DM patients whose TWEAK and Fn14 expressions in muscle tissue were examined received pathological diagnoses. Muscle biopsy is the most crucial test for establishing the diagnosis of PM/DM and can be used to exclude other neuromuscular diseases [1,39,40]. Therefore, the bias resulting from false disease diagnoses was reduced in our study.

However, we acknowledge that our study has some limitations. First of all, our sample size of PM/DM patients whose expression of TWEAK and Fn14 in muscle tissue was investigated was relatively small. This was partly due to our strict selection only of patients who had pathologically confirmed diagnoses. Another limitation of our study is that the expression of TWEAK mRNA in peripheral blood lymphocytes was not examined.

## Conclusions

In conclusion, our study showed that significantly high levels of TWEAK and Fn14 could be found in the muscle tissue of PM/DM patients, and Fn14 mRNA levels in muscle tissue positively correlated with muscle disease activity. Regardless of the precise mechanism, the TWEAK-Fn14 axis may probably be involved in the pathogenesis of PM/DM. Further understanding of TWEAK-Fn14 function in PM/DM may help to define therapeutic targets for PM/DM.

## Abbreviations

ANA: antinuclear antibody; BSA: bovine serum albumin; DM: dermatomyositis; EGF: epidermal growth factor; ELISA: enzyme-linked immunosorbent assay; Fn14: fibroblast growth factor-inducible 14; GAPDH: glyceraldehyde-3-phosphate dehydrogenase; H&E: hematoxylin and eosin; IBM: inclusion-body myositis; IIM: idiopathic inflammatory myopathies; ILD: interstitial lung disease; MAPK: mitogen-activated protein kinase; MYOACT: myositis disease activity assessment visual analog scales; PDGF: platelet-derived growth factor; PM: polymyositis; RT-PCR: reverse transcription polymerase chain reaction; TBA: tris-buffered saline; TNF: tumor necrosis factor; TWEAK: tumor necrosis factor-like weak inducer of apoptosis; VEGF: vascular endothelial growth factor.

## Competing interests

The authors declare no potential conflict of interest.

## Authors’ contributions

QLP and GCW contributed to study design, data collection, statistical analysis, interpretation of data and drafting the manuscript; XMS acquired the clinical data; XLT participated in the analysis of serum TWEAK levels; XL participated in study design, acquisition, analysis and interpretation of data. All authors were involved in drafting the article or revising it critically for important intellectual content, and all authors read and approved the final version.
